# Anti-Inflammatory Effects of Vitamin E in Response to *Candida albicans*

**DOI:** 10.3390/microorganisms8060804

**Published:** 2020-05-26

**Authors:** Silvana Barros, Ana Paula D. Ribeiro, Steven Offenbacher, Zvi G. Loewy

**Affiliations:** 1 Department of Periodontology, School of Dentistry, University of North Carolina, Chapel Hill, NC 27599, USA; silvana_barros@unc.edu (S.B.); steven_offenbacher@unc.edu (S.O.); 2 Department of Restorative Dental Sciences, College of Dentistry, University of Florida, Gainesville, FL 32610, USA; aribeiro@dental.ufl.edu; 3 Department of Pharmaceutical and Biomedical Sciences, Touro College of Pharmacy, New York, NY 10027, USA; 4 Department of Microbiology and Immunology, New York Medical College, Valhalla, NY 10595, USA

**Keywords:** in vitro, candidiasis, agonist

## Abstract

Oral mucositis, inflammation, and ulceration that occur in the oral cavity can manifest in significant pain. A formulation was designed to investigate the potential of vitamin E to ameliorate inflammation resulting from *Candida albicans* in cell-based systems. Human gingival fibroblasts and THP1 cells were stimulated with heat killed *C. albicans* and *Porphyromonas gingivalis* LPS (agonists). Unstimulated cells were included as controls. Cells were also simultaneously treated with a novel denture adhesive formulation that contains vitamin E (antagonist). The experimental conditions included cells exposed to the experimental formulation or the vehicle for 2 h for mRNA extraction and analysis, and cells left for 24 h under those experimental conditions for analysis of protein expression by ELISA. ssAffymetrix expression microarray pathway analyses demonstrated that the tested formulation exhibited a statistically significant (*p* < 0.05) inhibition of the following key inflammatory pathways: TLR 6, IL-1 signaling (IRAK, A20), NF-kappaB, IL-6 signaling (gp130, JK2 and GRB2), TNF signaling (TNF receptor) and Arachidonic acid metabolism (PLA2). Quantitative PCR array analysis confirmed the downregulation of key inflammatory genes when cells under adhesive treatment were challenged with heat killed *C. albicans*. PGE2 secretion was inhibited by the tested formulation only on THP1 cells after 24 h stimulation with *C. albicans*. These results suggest that the active formulation containing vitamin E acetate can modulate inflammatory responses, through anti-inflammatory actions as indicated by in vitro experimental conditions.

## 1. Introduction

Candidiasis, considered the most common human fungal infection [1,2], results from alterations in the balance between the host and *Candida* pathogen [3]. These alterations can comprise host systemic and local factors resulting from the presence of a removable denture prosthesis and related trauma, reduction of the salivary flow, and pH changes, and more broadly, systemic diseases and/or associated deficiencies of the immune system [4]. The result of an unbalanced relationship is the microorganism overgrowth and concomitant invasion of the mucosal tissue by this microorganism, resulting in inflammation and infection [4]. The manifestations of the disease are mucocutaneous and systemic infections [5,6]. Oropharyngeal candidiasis is frequently observed in denture wearers, immunosuppressed patients, i.e., HIV-infected patients and cancer patients [4,6].

*Candida albicans* is found among the normal commensal flora of mucosal surfaces and it is commonly isolated from the oral cavity. The prevalence of this fungi varies according to the type of population [3], and some studies showed that its prevalence in the oral cavity varies from 50%–75% of people who wear removable dentures [5,6]. Once adhered to epithelium, *C. albicans* initiate tissue invasion by two different mechanisms, induction of epithelial cell endocytosis and active penetration [7]. Both mechanisms result in cell damage and trigger an inflammatory response by the innate immune system involving neutrophils, monocytes/macrophages, natural killer (NK) cells, dendritic cells and non-hematopoietic cells, such as mucosal epithelial cells and fibroblasts [8,9,10]. The activation of the innate system produces different cytokines, chemokines, and other products such as antimicrobial peptides [8,10]. Some examples of cytokines and chemokines secreted in response to *Candida* infection are IL-1α, IL-1β, IL-8, IL-6, TNFα, GM-CSF, CX3CL1, and others [8,9,11,12,13,14,15,16]. 

The role of nutrients, especially vitamins, in participating in the immune response regulation has been demonstrated in studies in humans [17,18]. Vitamin C, a water-soluble compound, and vitamin E, a fat-soluble compound, are effective antioxidants involved in the maintenance of oxidative reactions and protection of membrane lipid peroxidation against reactive oxygen species (ROS) generated during an inflammatory response [17]. In vitro studies have shown that Vitamin C efficiently inhibited the lipopolysaccharide ( LPS)-challenged monocytes production of IL-6 and TNF and lymphocytes production of IL-2 [18]. The authors speculated that this inhibition might be due to the downregulation of NF-kB or T-cell induced apoptosis-signaling pathways [18]. Vitamin E was shown to be able to block *E. coli* LPS-induction of inducible nitric oxide synthase (iNOS), COX-2, and NF-kB expression in monocytes [19].

There are no studies evaluating the anti-inflammatory effects of vitamin E in inflammation in response to *C. albicans*. The overall goal of this study was to explore the potential anti-inflammatory properties of a novel dental adhesive formulation, containing vitamin E as its principal active component for candidiasis. The rationale was to explore the potential of a denture adhesive to deliver vitamin E. The novel denture adhesive formulation would continue to provide functionality for food occlusion while at the same time reducing inflammation. The present study was developed using two different human cell types, monocytes and fibroblasts, challenged with heat killed *C. albicans* and *P. gingivalis* LPS. The effects of this novel adhesive formulation were examined by gene expression patterns of those cells exploring the whole transcriptome using Affymetrix arrays and confirmed by quantitative PCR array (RT^2^ arrays) and protein secretion by ELISA.

## 2. Materials and Methods

### 2.1. Cell Culture

The Human Gingival Fibroblasts (ATCC#CRL-2014) were propagated in Dulbecco’s Modified Eagle’s medium with 4 mM L-glutamine adjusted to contain 1.5 g/L sodium bicarbonate and 4.5 g/L glucose, supplemented with 10% of fetal bovine serum. The THP-1-monocytic cell line (ATCC#TIB-202) was propagated in RPMI 1640 medium with 2 mM L-glutamine adjusted to contain 1.5 g/L sodium bicarbonate, 4.5 g/L glucose, 10 mM HEPES and 1.0 mM sodium pyruvate and supplemented with 0.05 mM 2-mercaptoethanol and 10% fetal bovine serum. 

### 2.2. Agonists

*Candida albicans* strain 28366, originally isolated from a human mouth, was purchased from American Type Culture Collection (ATCC, Rockville, MD, USA). The yeast was routinely propagated using Sabouraud dextrose agar at 30 °C. Stationary phase organisms were prepared by growth for 18 h at room temperature in Sabouraud dextrose broth at 30 °C. After centrifugation, the pellet containing the yeast cells was diluted with 10 mM sodium phosphate buffer (PBS) and the *C. albicans* concentration was determined photometrically at OD 660 as 2 × 10^8^ CFU/mL. *C. albicans* were heat-killed at 80 °C in a water bath for 1 h. A final concentration of 1 × 10^7^ CFU/mL of heat-killed *C. albicans* was used for cell stimulation.

*P. gingivalis* Ultrapure LPS (San Diego, CA) stock solution was prepared in endotoxin-free water and added to the medium at a final concentration of 0.3 μg/mL.

### 2.3. Antagonists

This in vitro study tested the potential anti-inflammatory activity of the Healthy Hold Complex formulation used in the PoliGrip Gum Health^®^. This formulation containing vitamin E acetate was tested in comparison to the vehicle with no actives (Table 1).

### 2.4. Experimental Conditions

A total of 0.2 g of each sample was used per well for each condition tested in 24-well-plates. Formulations were saturated with 1 mL of pre-warmed complete culture media in each well of cell culture media for 2 h at 37 °C and then the inserts were placed overnight in the 24 well-plates to support the growth of the cells. Cells were plated at a density of 5 × 10^5^ cells/well for THP1 cells and 5 × 10^4^ cells/well for fibroblasts. Cells were stimulated with either *P. gingivalis* LPS or heat killed *C. albicans* or kept unstimulated. To test antagonist formulations, cells were incubated with agonists for either 2 h for mRNA expression or 24 h for cytokine release into the media. After 2 h of treatment (stimulation), fibroblasts and THP1 cells were harvested for mRNA extraction (Qiagen Inc., Germantown, MD, USA). Cells showed from 87% to 92% viability after the stimulations.

### 2.5. Gene Expression Analysis

#### 2.5.1. Affymetrix Microarrays

Cells were processed for mRNA extraction and gene expression profile mapping. RNA was isolated from cells via standard molecular biology protocols. Gene expression profiling was performed using Affymetrix (Affymetrix Inc., Santa Clara, CA, USA) recommended procedures. RNA quality was assessed by a Bioanalyzer 2100 with a quality control cut-off of RIN of 8 or higher. Gene chip targets were synthesized from the RNA using Affymetrix target synthesis procedures. Targets were hybridized to gene chips followed by a series of wash and stain protocols. Each gene chip was scanned using photoluminescence. Affymetrix GeneChip Microarray Suite 5.0 software, Thermo Fisher Scientific, Santa Clara, CA. was used for scanning, and basic analysis. This permits the creation of a normalized feature dataset. Identification of disease-specific gene expression profiles was performed using the Partek Genomics Suit. Pathway analyses were performed using Ingenuity Pathway Analyses (IPA).

#### 2.5.2. Pathway-Focused Gene Expression: RT^2^ Profiler PCR Arrays

RNA was reverse transcribed to cDNA using the Omniscript RT kit (Qiagen Inc.), cDNA levels and pro-inflammatory mediator molecules were measured using RT2 pathway arrays and the ABI Prism 7500 Sequence Detection System (Applied Biosystems, Foster City, CA, USA). The fold change for each analysis was calculated using the values of non-stimulated samples as calibrator by means of 2∆CT method for mRNA gene expression for human common cytokines (RT2 Profiler™ APH-021).

### 2.6. ELISA

To complement the data obtained from the gene expression experiments, culture supernatants were analyzed by ELISA. Briefly, supernatant (medium) was added to 96-well plates coated with 1 μg/mL monoclonal antibodies (PGE_2_). A secondary biotinylated antibody specific for the cytokine of interest was added as the detecting antibody, followed by a streptavidin-horseradish peroxidase conjugate (enzyme). Tetramethylbenzidine was used to bind enzyme and produce color. The reaction was stopped by the addition of 2 N hydrochloric acid, and the optical density was determined using a VMax microplate reader (Molecular Devices, Palo Alto, CA, USA) at 450 nm against a standard curve based on known concentrations of the recombinant cytokine.

## 3. Results

### 3.1. Gene Expression Array by Affymetrix

**Figure 1:**
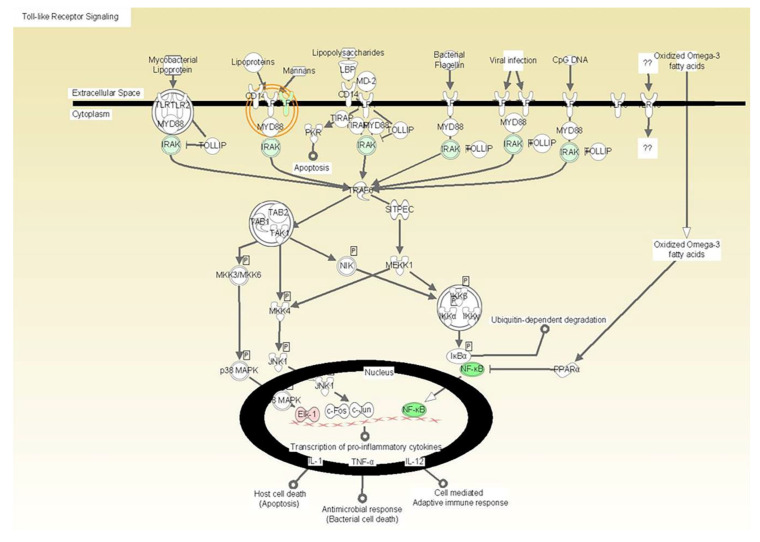
The illustration depicts the localization of the gene products by cellular compartmentalization including extracellular space, cytoplasm, and nucleus and indicates the downregulated expression of Toll-like receptor (TLR) signaling molecules as an effect of vitamin E formulation treatment on heat killed *C. albicans*-stimulated fibroblasts with associated p-values. All gene products highlighted in green are significantly down-regulated. The downregulated expression of Toll-like receptor (TLR) signaling molecules as an effect of vitamin E formulation treatment on heat killed *C. albicans*-stimulated fibroblasts with associated p-values. All gene products highlighted in green are significantly downregulated.

**Figure 2:**
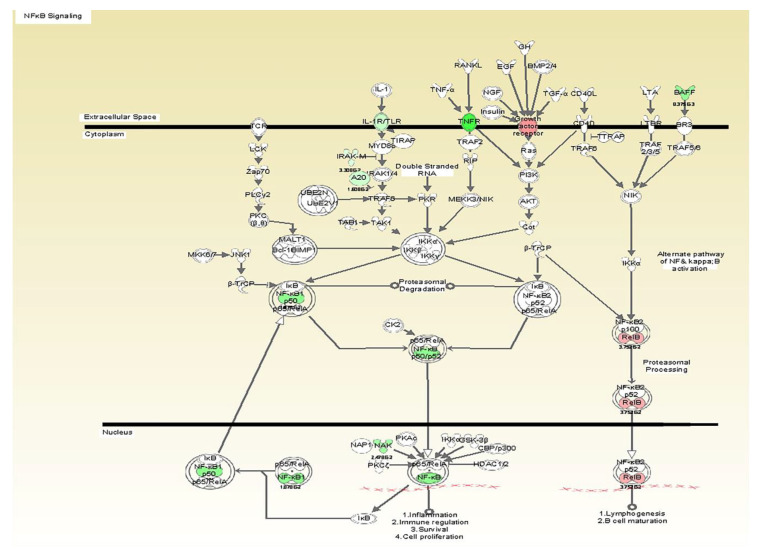
The illustration depicts the localization of the gene products by cellular compartmentalization and indicates the downregulated expression of NF-kappaB signaling pathway as an effect of vitamin E formulation treatment on heat killed *C. albicans*-stimulated fibroblasts with associated *p*-values. All gene products highlighted in green are significantly down-regulated.

**Figure 3:**
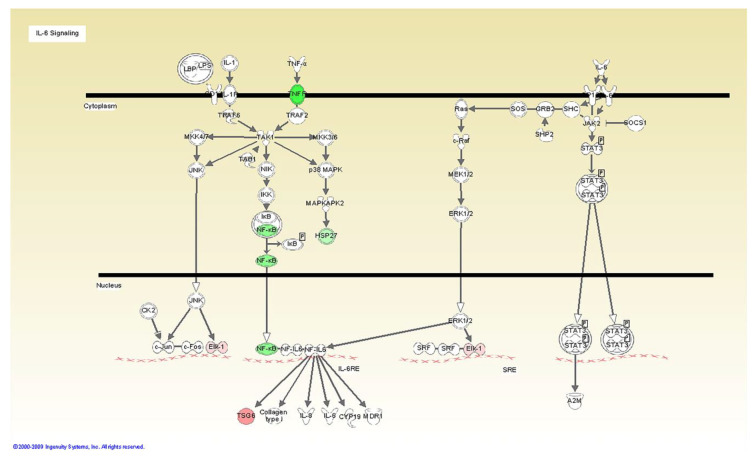
TNF pathway inhibition in HGF in response to *C. albicans* agonist when treated with vitamin E formulation. All gene products highlighted in green are significantly down-regulated.

A series of arrays were analyzed with several internal controls. Arrays were run for each agonist and cell type. Statistical significance was assessed by a fold change, regression analysis determining whether the slope (beta coefficient) was significantly greater than zero. No individual genes passed the FDR 0.05 criteria; however several pathways were statistically significant by Ingenuity Pathway Analyses. The results presented below reflect the significant pathways that were activated by the active formulations on the fibroblasts and THP1 cells (Table 2). The tested formulation containing vitamin E induced a dose dependent inhibition of the TLR6 pathway including the Mannan receptor TLR6, IRAK, and NF-kappaB (Figure 1, Figure 2 and Figure 3).

### 3.2. Gene Expression-RT2 Profiler PCR Arrays

**Figure 4:**
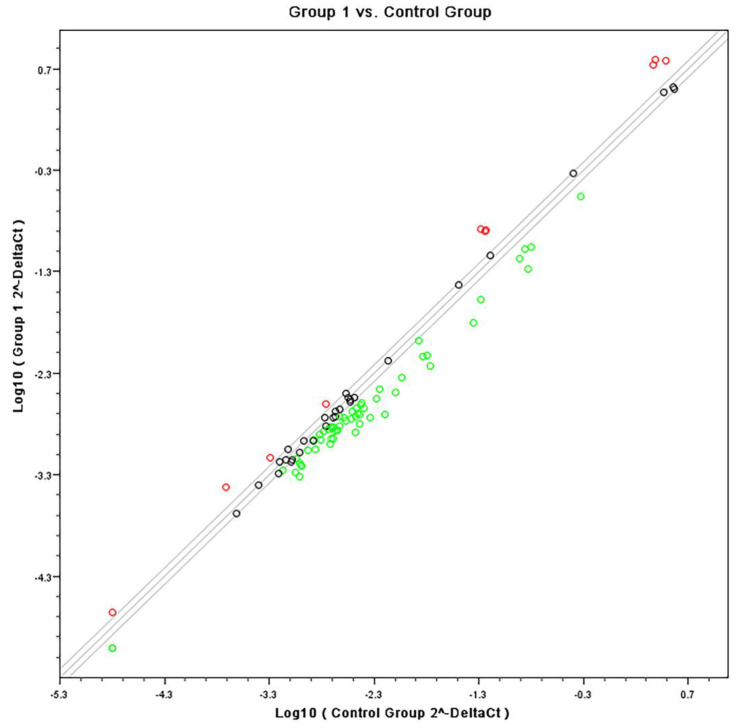
Scatter plot showing the comparison of gene expression levels between the common inflammatory gene expression data set on Fibroblasts simultaneously treated with tested formulation and stimulated with *C. albicans* (group 1) versus the control (vehicle) treated cells under *C. albicans* stimulation.

**Figure 5:**
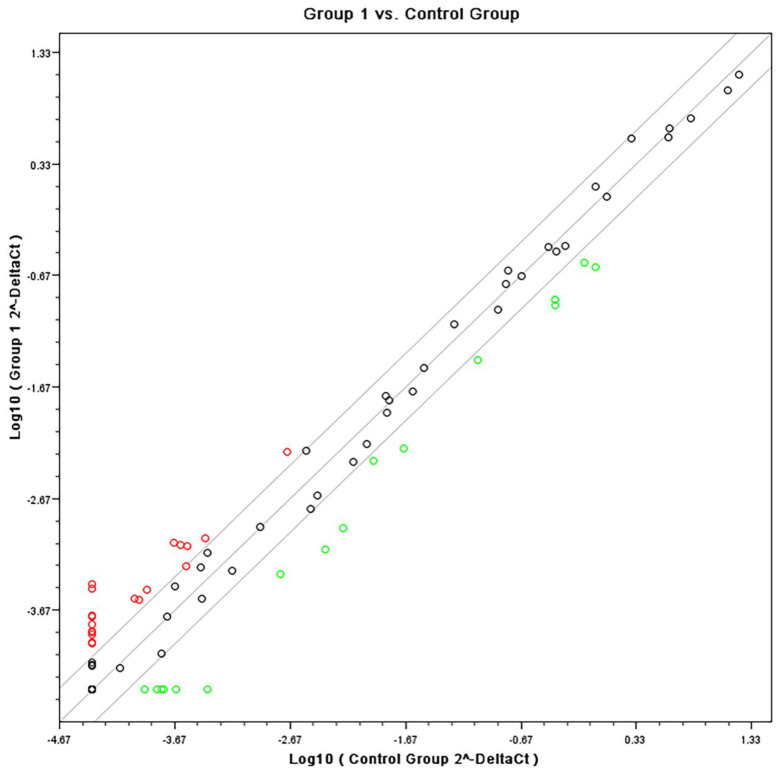
Scatter plot showing the comparison of gene expression levels between the data set on common inflammatory gene expression data set on THP1 cells simultaneously treated with tested formulation and stimulated with *C. albicans* (group 2) versus the control (vehicle) treated cells under *C. albicans* stimulation.

Quantitative PCR confirmed specific pathways that were identified by the Affymetrix analyses using SABiosciences RT profiler pathway arrays for “Common Cytokines”. The data obtained from gene expression showed a down regulation of inflammatory genes for both cell types when stimulated with heat-killed *C. albicans*. Figure 4 and Figure 5 are scatter plots showing the comparison of gene expression levels between the data sets on fibroblasts and monocytes respectively, treated with the experimental adhesive and stimulated with *C. albicans* (group 1) versus the control (vehicle) treated cells under *C. albicans* stimulation. Three solid lines are shown diagonally across the scatter plot. The middle green is the identity line, or the x = y line. Data points on this line represent genes that are expressed at the same level in both datasets. The other two lines delineate genes with at least a two-fold change in intensity value (up regulated in red or down regulated in green) in one of the datasets.

RT^2^ cytokine pathway gene array differential expression in THP-1 cells when challenged with *C. albicans* in the presence of the active formulation is shown in Table 3. A series of arrays were analyzed with several internal controls. The 3 arrays were run for each agonist and cell type. Table 4 shows the up-down regulation of gene expression of interferons in THPs and Fibroblasts of the experimental group when compared to the vehicle. Table 5 shows the downregulation of TNF α measured by a PCR array on Human Gingival Fibroblasts.

### 3.3. ELISA Results

**Figure 6:**
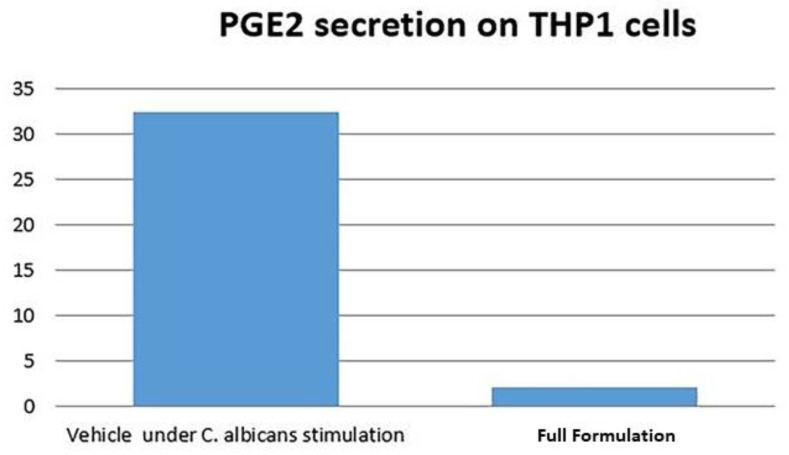
Protein assay by ELISA: PGE2 secretion (ng/mL) on THP1 cells after 24 h stimulation with *C. albicans*.

The data obtained from ELISA analysis showed that PGE2 secretion was inhibited by the tested formulation only on THP1 cells after 24 h stimulation with *C. albicans* (Figure 6).

## 4. Discussion

In this study, we examined the anti-inflammatory and antioxidant effects of a formulation with vitamin E acetate, ethyl paraben, and methyl paraben in fibroblasts and monocytes challenged with *C. albicans* and *P. gingivalis* LPS. Progress in development of multi-targeted drugs for regulating inflammatory diseases has brought interest in vitamin E, an antioxidant lipid [20,21,22,23]. Vitamin E, an essential nutrient with powerful antioxidant activity, is the resultant mixture of two classes of compounds, tocopherols and tocotrienols. The potential of vitamin E to regulate inflammation has been evaluated by investigating the effects of tocotrienol, a derivative of vitamin E, on the LPS- induced inflammatory response in THP-1 cells. The authors showed that tocotrienol could efficiently inhibit LPS-induced NO generation, PGE2 production, cytokine (TNF-a, IL-4, and IL-8) secretion, iNOS, COX-2, and NF-kB expression in monocytes [19]. Tocopherol, the most common class of vitamin E, has also been shown to regulate leukocyte recruitment during allergic lung inflammation in vivo. Moreover, α-tocopherol supplementation reduces, and γ-tocopherol elevates this recruitment [23,24]. Due to the potential to downregulate the inflammatory response, we aimed to investigate the anti-inflammatory effects of vitamin E present in a novel adhesive formulation using an in vitro model of candidiasis.

In our study, we found inhibition of key inflammatory markers after challenge with *C. albicans* in both tested cell types. The THP-1 monocytes represent an important cell line in the immune innate response to fungal infection. It also possesses a homogeneous genetic background resulting in less variability in gene expression profiles [25]. The monocytic cells, THPs, have been used before to study chemokine and cytokine production in response to fungal cells and cell-wall components [13,14], and to evaluate changes in host gene expression [25]. Those studies showed that *Candida* cell wall components and live cells upregulated the gene expression and secretion of TNF-α, IL1-B and IL-8 and H_2_O_2_ release. Our findings indicate that the vitamin E containing formulation could significantly downregulate the pathways of the interleukins: IL1A, IL1B, IL6, Il8, Il10, and Il12. A similar blockade of inflammatory markers was found by Wu et al. utilizing the THPs and emphasizing the effect of tocotrienol in the regulation of Il1b, Il8, and TNF α in monocytic cells.

For the tested fibroblasts, we observed that the formulation suppressed gene expression of important cytokines commonly involved with the response to pathogens, such as IFNs and TNF. The role of oral fibroblasts in candidiasis has not been widely studied. During *Candida* invasion, the mucosal barrier is penetrated, and the connective tissue is exposed to the fungal virulence factors such as adhesins and hydrolytic enzymes, such as lipase, phospholipase, and proteinases [26]. In this way, oral fibroblasts may also play a role in the innate immune response to fungal infection with the production of inflammatory markers as reported by Ohta et al., who showed that live and heat-killed *C. albicans,* as well as non-albicans strains, caused an increase in CX3CL1 mRNA and protein expression in human immortalized oral fibroblasts [14].

Regarding the in vitro model, we used heat killed *C. albicans* and *P. gingivalis* LPS to challenge fibroblasts and monocytes in order to induce an inflammatory response. The heat killed *Candida* has been used in various studies to evaluate the live yeast/hyphae potential to promote upregulation of certain cytokines and chemokines [27,28]. It is known that live *Candida* invade the cell by active penetration and endocytosis which will result in cell damage and induction of an inflammatory response [7]. However, *Candida* cell wall components, such as mannan, have been shown to stimulate macrophages and epithelial cytokine responses [27,29,30], and to upregulate TLR4 expression, suggesting that cell injury or the exposure of deeper epithelial layers to *C. albicans* cells is not required to trigger PMN-mediated TLR4 upregulation [28].

Although heat killed *Candida* and LPS can trigger an inflammatory response, our data showed that LPS stimulation induced a stronger inflammatory response for both tested cell types while heat killed *C. albicans* induced a moderate inflammatory upregulation of many of the targeted genes measured by RT^2^ expression array. Moreover, the tested adhesive formulation provided only modest suppression of the inflammatory response elicited by LPS, in comparison to a more significant inhibition of inflammatory markers in the presence of heat killed *C. albicans*. Joualt et al. found that although *Candida* phospholipomannan could induce TNF-α production by THP-1 monocytes, the amounts of this cytokine were limited when compared to LPS stimulation [31]. By using epithelial cells to test different compounds from microbes on the induction of pro-inflammatory cytokines and chemokine expression, Pivarcsi et al. found that both, LPS and heat killed *Candida,* induced the expression of IL-8 and TNF-α, although the treatment with *C. albicans* resulted in a markedly lower induction when compared to the Gram negative cell wall compound [32].

Affymetrix microarray results showed that although no individual genes passed the FDR 0.05 criteria, several pathways were statistically significant by IPA™. This data might be reflecting a limitation of this in vitro model, which could not detect significant changes in gene expression. The data also showed that the tested formulation induced a dose dependent inhibition of the TLR6 pathway including the Mannan receptor TLR6, IRAK, and NF-kappaB. Different types of pattern-recognition receptors have been reported to participate in the recognition of *Candida* by the innate host defense including Toll-like receptors (TLR), C-type lectin-receptor (CLR) and NOD-like receptor [28]. TLR2 and TLR4 have been implicated in host defense against *Candida*; however, the majority of these studies used TLR recognition by epithelial and myeloid cells [28,33]. A recent study demonstrated that a wild-type strain of *Candida* induced TLR2, TLR4, TLR6, and TLR9 gene expression activation in the epithelial cells showing that different receptors may play important roles in candidiasis [34]. TLR6 has been shown to form heterodimers with TLR2 [35] and through heterodimerization, this receptor complex can recognize bacterial diacyl lipopeptides and lipoteichoic acid [36,37]. Netea et al. showed that recognition of *C. albicans* by TLR6 modulated the balance between Th1 and Th2 cytokines, with TLR6 knockout mice displaying a more pro-inflammatory cytokine profile, characterized by increased IFN-g production and reduced IL-10 [34].

Another pathway that was significantly inhibited was NF-kB activation. We also detected downregulation of IRAK, which is a key adaptor protein involved in the TLR signaling and interleukin signaling that leads to activation of NF-kappaB. The activation of this protein complex happens in response to tobacco, stress, dietary agents, obesity, alcohol, infectious agents, irradiation, and other environmental stimuli [38,39]. Once NF-kB is triggered, it is a master transcriptional control factor that has broad inflammatory activities increasing the expression of IL-1, TNF, IL-6, COX-2, and many cascading pro-inflammatory signals. Therefore, the inhibition of this pathway may account for the broader anti-inflammatory activities of the vitamin E containing formulation in response to *C. albicans* challenge. Some studies reported the ability of vitamin E compounds to suppress NF-kB activation [19,40]. Ahn et al. found that γ-tocotrienol completely suppressed TNF-induced NF-kB activation in human chronic myeloid leukemia cells [41]. Moreover, Wu et al. showed that tocotrienol derived from palm oil possess potent anti-inflammatory activity through blocking NF-kB activation and selectively inhibiting the COX-2 expression. In this way, the activity of the tested formulation is a result of its ability to downregulate TLR6 and NF-kB. Although the tested adhesive was able to downregulate TLR and NF-kB pathways, it is important to note that this broad activity was not evident with LPS as the primary agonist.

The tested adhesive also exhibited inhibition of TNF α signaling and the mediator of chronic inflammation IL-6, consistent with the PCR data. These signaling molecules are also under NF-kB regulation supporting the concept that the formulation has broad anti-inflammatory activities. Finally, regarding ELISA analysis, we observed a 6 fold reduction of PGE2 secretion for the formulation group when compared to control. The downregulation of this protein by the vitamin E formulation is an important indicator of anti-inflammatory effect in cells, which is consistent with the results presented by Wu et al. These authors observed that the co-treatment of THP-1 monocytes with LPS plus tocotrienol at various concentrations significantly suppressed LPS-induced PGE2 production.

## 5. Conclusions

In the in vitro models used in this study, the formulation containing vitamin E acetate has been shown to have broad anti-inflammatory activity against *C. albicans*, due to the suppression of cascading activities of NF-kB; although, it was not as effective in suppressing the strong inflammatory stimulus elicited by *P. gingivalis* LPS. Since *C. albicans* represents a primary challenge in oral stomatitis, additional clinical studies are required to investigate the clinical anti-inflammatory efficacy of the formulation.

## Figures and Tables

**Table 1:** Healthy Hold Complex Formulation.

	Test Formulation	Control (Vehicle)
Carboxymethyl cellulose (CMC)	yes	yes
Mineral Oil, Light	yes	yes
Petrolatum Blend	yes	yes
Double Salt	yes	yes
Vitamin E Acetate	yes	0.00%
Ethyl Paraben	yes	0.00%
Methyl Paraben	yes	0.00%

**Table 2:** Inflammatory pathways exhibiting downregulation of key molecules.

-TLR 6 synthesis and signaling [processing carbohydrates and mannans associated with fungi]
-IL-1 synthesis and signaling [IRAK3, A20]
-NF-kappaB: A master transcriptional regulator that enhances the expression of multiple inflammatory mediators.
-IL-6 synthesis and signaling [gp130, JK2 and GRB2]
-TNF synthesis and signaling [TNF receptor]

**Table 3:** Fold changes in inflammatory markers expression levels in THP-1 cell when challenged with *C. albicans* in presence of the active formulation indicating its effects on the regulation of biomarker levels in monocytes.

Gene Symbol	Adhesive + *C. albicans* THP1
IL1A	−1.5751
IL1B	−1.0392
IL6	−1.3753
IL8	−2.8064
IL10	−1.5208
IL12A	−1.7109

**Table 4:** Up- downregulation (comparing to Vehicle + *C. albicans* = 1.0) of Interferons in THPs and Fibroblasts.

Gene Symbol	THP1 Full Formulation + *C. albicans*	Fibroblasts Full Formulation + *C. albicans*
IFNA1	1.3822	
IFNA2	−1.1669	
IFNA4	−1.8824	
IFNA5	−1.619	
IFNA8	−1.619	
IFNB1	1.1665	2.6224
IFNG	−1.2247	3.9441

**Table 5:** Downregulation of TNF showed by PCR array on Human Gingival Fibroblasts.

Gene Symbol	Fibroblasts P&P *C. albicans*
TNFSF11	−4.1403
TNFSF12	−1.1864
TNFSF13	1.7268
